# Non-linear relationship between the children’s dietary inflammatory index and asthma risk: identifying a critical inflection point in US children and adolescents

**DOI:** 10.3389/fnut.2025.1538378

**Published:** 2025-03-21

**Authors:** Junyu Xu, Meiping Zhu, Yi Shi, Qian Chen, Yan Zhu

**Affiliations:** The First Affiliated People's Hospital of Huzhou University, Huzhou, Zhejiang, China

**Keywords:** children, adolescents, dietary inflammation, food intake, asthma risk

## Abstract

**Background:**

Asthma, a chronic respiratory disease, is influenced by diet, which plays a key role in its onset and progression. The Children’s Dietary Inflammatory Index (C-DII) measures how diets impact inflammation in children and adolescents (6–19 years). The C-DII is a metric that quantifies the inflammatory potential of diet, with higher scores indicating more pro-inflammatory diets and a scoring range from −6.25 to 6.02. This study investigates the association between C-DII and asthma prevalence in US children and adolescents.

**Methods:**

Data from the National Health and Nutrition Examination Survey (NHANES) 2013–2018 and 2021–2023 were utilized. Data from participants aged 6–19 years who completed dietary interviews and provided asthma-related information was included. The C-DII was calculated using 24-h dietary recall data, and data were categorized into quartiles. Asthma diagnosis was based on self-reported doctor diagnosis and current asthma status. Multivariable logistic regression, smooth curve fitting, threshold benefit analysis, and Restricted Cubic Spline (RCS) analyses were performed to evaluate the relationship between C-DII and asthma prevalence.

**Results:**

Data from 6,523 children and adolescents aged 6–19 years were analyzed. The median C-DII score was-0.026, and asthma prevalence was 18.63%. A U-shaped relationship was observed between C-DII and asthma prevalence, with the lowest risk at a C-DII score of-0.99. Subgroup analyses revealed variability in the association between C-DII and asthma across demographic groups. Age-based analysis indicated significant interaction (*p* = 0.047), with the weakest association observed in the 17–19 years age group. Ethnicity showed significant differences, particularly in Mexican-American (OR = 0.83, 95% CI: 0.70–0.97) and Non-Hispanic Black (OR = 1.56, 95% CI: 1.36–1.80) subgroups.

**Conclusion:**

This study underscores a significant non-linear association between C-DII and asthma prevalence in US children and adolescents, emphasizing the importance of balanced dietary patterns in mitigating asthma risk. Future longitudinal studies are warranted to confirm these findings and explore causal pathways.

## 1 Introduction

Characterized by airway hyperresponsiveness and inflammation, asthma is a chronic respiratory disorder that manifests with symptoms such as episodic wheezing, coughing, and breathlessness, which affects a significant proportion of the global pediatric population (1, 2). In the United States alone, approximately 7.7% of children and adolescents under the age of 18 are diagnosed with asthma, making it one of the most common chronic diseases in this demographic (3). Globally, asthma affects an estimated 300 million people, with increasing prevalence noted over the past few decades (4, 5). This upward trend highlights the urgent need to identify modifiable factors associated with its onset and progression. Recent evidence suggests that diet play a crucial role in the prevention and management of asthma, offering promising avenues for intervention in at-risk populations (6–8).

Inflammation of a persistent but mild nature is extensively associated with the development of asthma, with a particular emphasis on its impact in the pediatric population (9). Emerging evidence suggests that dietary patterns and specific nutrients can exert pro-or anti-inflammatory effects, potentially influencing asthma risk and severity through mechanisms such as modulation of the intestinal microbiota, oxidative stress, and immune system regulation (10, 11). Diets high in refined sugars, saturated fats, and processed foods promote systemic inflammation and increase the risk of chronic diseases such as cardiovascular disease, diabetes, and arthritis (12). In contrast, diets rich in fruits, vegetables, whole grains, and omega-3 fatty acids contain bioactive compounds like antioxidants and fiber that mitigate inflammation and enhance immune function (13). Recent studies and a systematic review highlight the role of dietary patterns in modulating inflammation and improving health outcomes in individuals with chronic inflammatory conditions (14). To quantify the inflammatory potential of diets, the Dietary Inflammatory Index (DII) was developed, incorporating the complex interactions of multiple nutrients and dietary components into a single composite score (15). A modified version, the Children’s Dietary Inflammatory Index (C-DII), is specifically tailored for use in pediatric populations and has a scoring range from −6.25 to 6.02 (16). Studies have demonstrated that higher DII scores are associated with increased markers of systemic inflammation and worse asthma outcomes in adults (17). However, while these associations are well-established in adults, the relationship between C-DII and asthma risk or severity in children remains underexplored, underscoring the need for further investigation in this demographic.

To address the aforementioned research gaps and provide further insights, this study aimed to investigate the association between the C-DII and asthma prevalence among US children and adolescents.

## 2 Methods

### 2.1 Data source and study participants

Data were obtained from the National Health and Nutrition Examination Survey (NHANES), a nationally representative program assessing health and nutritional status. NHANES is an investigative initiative that evaluates the health and nutritional conditions of both adult and pediatric populations in the United States by employing a blend of questionnaires and medical assessments. Protocols for this research were sanctioned by the Institutional Review Board of the National Center for Health Statistics, and written consent was obtained from all participants (36).

This analysis utilized data from the 2013–2018 and 2021–2023 cycles of NHANES, ensuring comprehensive and representative findings for the US pediatric population. Children under 6 were excluded due to differences in dietary patterns and nutritional needs, which may affect asthma prevalence differently.

Eligibility for the study, which drew participants from NHANES conducted over the periods 2013–2018 and 2021–2023, was determined by the following criteria: (i) participants were aged 6 to 19; (ii) they completed dietary interviews (We used data averaged from 2 days to maximize sample size and representativeness. Extreme values were defined as energy intake exceeding ±3 standard deviations from the sample mean and excluded from analysis.); and (iii) they provided asthma-related information through medical questionnaires.

**Figure 1:**
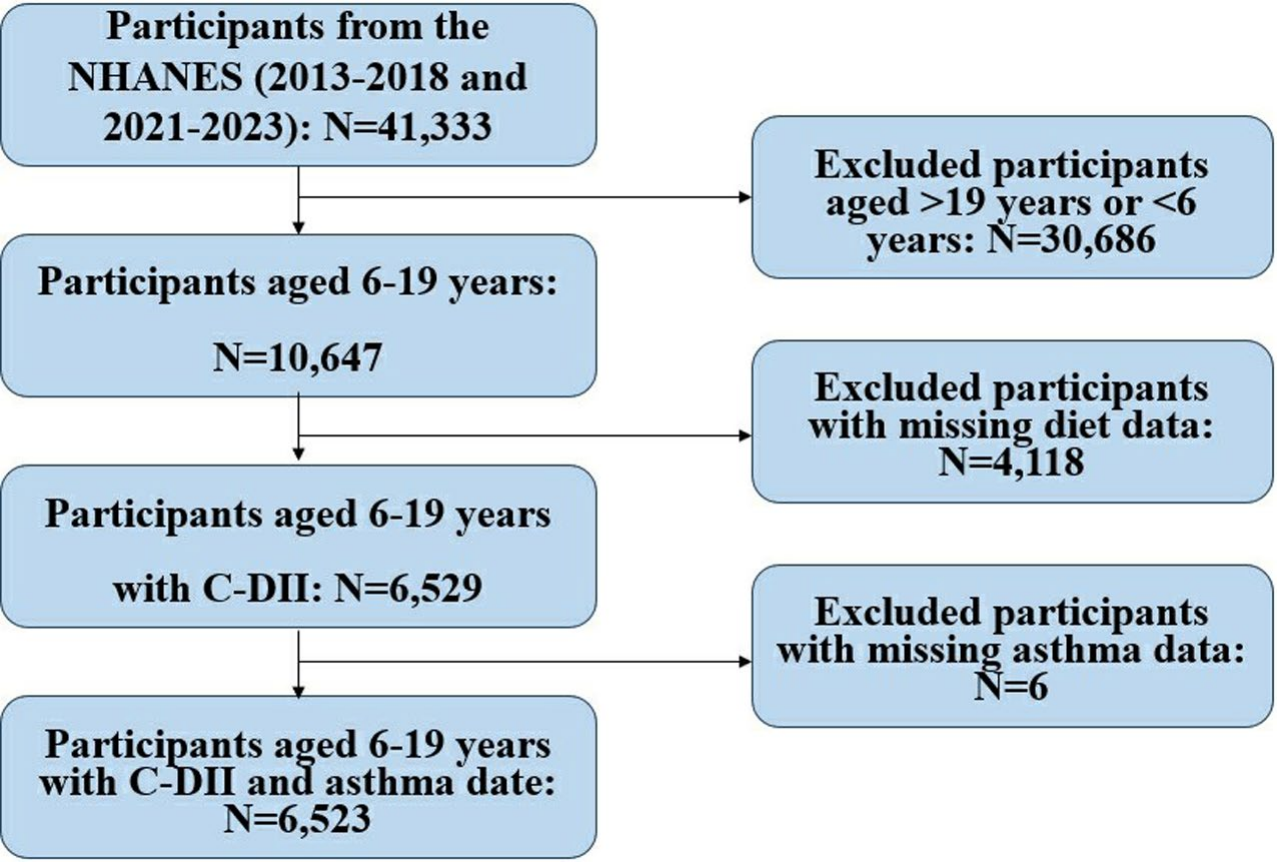
Flowchart of participant inclusion/exclusion.

The study utilized NHANES data and included 41,333 participants. After excluding individuals outside the 6–19 age range (*N* = 30,686), those with missing dietary data (*N* = 4,118), and missing asthma data (*N* = 6), a final sample of 6,523 children and adolescents was analyzed to investigate the relationship between the C-DII and asthma. They were included in the final dataset for analysis (Figure 1).

### 2.2 C-DII assessment

C-DII aligns closely with that of its adult counterpart, the DII. While the adult DII incorporates 45 dietary components, the C-DII employs a subset of 25 parameters, reflecting key differences in dietary patterns between children and adults (18). The C-DII is calculated using 24-h dietary recall data, focusing on nutrient intake. Specifically, it incorporates the following 25 nutrients: carbohydrates, proteins, total fats, alcohol, dietary fiber, cholesterol, saturated fatty acids, monounsaturated fatty acids, polyunsaturated fatty acids, niacin, thiamin, riboflavin, vitamin B12, vitamin B6, iron, magnesium, zinc, selenium, vitamin A, vitamin C, vitamin E, folate, beta-carotene, energy, and total sugars. Each nutrient is assigned a dietary inflammatory score based on its pro-or anti-inflammatory properties.

The validity of the C-DII has been well-established, showing a significant positive correlation with biomarkers of chronic inflammation and oxidative stress (19). This relationship has been consistently observed in both pediatric and adolescent populations. The calculation of the C-DII involves three key steps. First, a Z-score is computed by subtracting the global mean daily intake from an individual’s reported intake and dividing this value by the global standard deviation. Second, to adjust for skewness and ensure a normal distribution, these Z-scores are converted into percentiles, doubled, and offset by subtracting one. Finally, the adjusted percentiles are multiplied by the nutrient-specific inflammatory scores, and the resulting values are summed to calculate the overall C-DII score. We ensured the reliability of the results through multiple data checks (e.g., random sampling to verify calculation consistency).

In this study, participants were divided into four quartiles based on their average C-DII scores: the first quartile (−6.25 ≤ C-DII ≤ −1.95), the second quartile (−1.95 < C-DII ≤ −0.03), the third quartile (−0.03 < C-DII ≤ 1.90), and the fourth quartile (1.90 < C-DII ≤ 6.02). This stratification facilitates a detailed analysis of the association between dietary inflammatory potential and various health outcomes in pediatric populations.

### 2.3 Asthma

Asthma diagnosis was ascertained from the US National Health Interview Survey questionnaire section within NHANES. Participants were identified as having current asthma if they affirmed two queries: ‘Has a doctor ever diagnosed you with asthma?’ and ‘Do you currently have asthma? (Currently experiencing symptoms.)’. Participants were classified as asthmatic only if both conditions were present. Proxy responses were accepted for participants under 16. Controls were those without current asthma, who negated these questions.

### 2.4 Covariates

Covariates including age, sex, race/ethnicity, poverty income ratio (PIR), and BMI were considered. A higher PIR indicates a higher socioeconomic status and lower poverty, while a lower PIR reflects greater poverty and lower socioeconomic status. Data for these variables are publicly accessible via the NHANES official website.^1^

### 2.5 Statistical analyses

The 24-h dietary recall data, which accounts for non-response and recall day, was utilized with corresponding sample weights for logistic regression analysis to reflect the US adolescent population, aligning with NHANES’s multistage sampling design. Continuous variables are presented as weighted means with 95% confidence intervals (CI), while categorical variables are expressed as weighted proportions.

Multivariable logistic regression was employed to assess the association between the C-DII and asthma, with outcomes expressed as odds ratios (OR) and 95% confidence intervals. Three models were estimated: Model 1 (unadjusted), Model 2 (adjusted for age and gender), and Model 3 (further adjusted for ethnicity, BMI, and PIR). All models incorporated sampling weights. Confounding was assessed using a change-in-estimate criterion (<10% threshold), and interactions were tested with multiplicative terms and Wald tests. Consequently, we performed smooth curve fitting to analyze the threshold effects and identify the most significant inflection point.

Additionally, we performed subgroup analyses on the fully adjusted model across various demographic and socioeconomic strata: gender (males and females), age (6–9, 10–13, 14–16, and 17–19 years), ethnicity (Mexican American, other Hispanic, Non-Hispanic White, Non-Hispanic Black, and other races), PIR, (≤1.3, poor/low-income; 1.3–3.5, middle-income; and > 3.5, economically well-off), and BMI (Underweight, Normal, Overweight, and Obese). BMI categories are based on the WHO 2006/2007 classification, using BMI-for-age Z-scores by age and sex.

Finally, we employed Restricted Cubic Spline (RCS) analysis to investigate the non-linear association between the C-DII and asthma.

Statistical power calculations were not prespecified; sample size was constrained by available data. Analyses were conducted using Python 3.11.8, with descriptive statistics for all participants employing two-tailed tests at a significance threshold of *p* < 0.05.

## 3 Results

### 3.1 Study population and weighted baseline characteristics

The study included 6,523 children and adolescents aged 6–19 years, with a median age of 12 years. The gender distribution was nearly balanced (50.57% females, 49.43% males). The majority of participants were aged 10–13 years (29.80%), followed by 6–9 years (28.91%), 14–16 years (22.09%), and 17–19 years (19.19%). Racial/ethnic composition was 30.19% Non-Hispanic White, 22.50% Non-Hispanic Black, 20.27% Mexican American, 10.56% Other Hispanic, and 16.48% other races (including multi-racial). The median PIR was 1.85, with 37.01% ≤1.3 (poor/low-income), 43.17% 1.3–3.5 (middle-income), and 19.82% >3.5 (economically well-off). BMI categories included 47.22% normal weight, 29.07% obese, 22.59% overweight, and 1.12% underweight. The median C-DII score was-0.026, and asthma prevalence was 18.63%.

The study population’s baseline characteristics were compared across the four groups of C-DII. Demographic variables are reported with *p*-values indicating statistical differences.

Gender distribution showed no significant difference across C-DII groups (*p* = 0.056), with 50.57% females and 49.43% males. Age distributions were also not significantly different across groups, with the following breakdown: 6–9 years (28.91%, *p* = 0.281), 10–13 years (29.80%), 14–16 years (22.09%), and 17–19 years (19.19%). These data provide a demographic overview, showing that both gender and age distributions were generally similar across C-DII groups. However, significant associations were found between C-DII and race/ethnicity (*p* = 0.001), PIR (*p* = 0.009), and BMI (*p* = 0.012), indicating potential differences in these covariates across groups (Table 1). There was no significant evidence of gender or age differences that could influence the relationship between C-DII and asthma, though race, PIR, and BMI showed notable variation.

**Table 1:** Weighted baseline characteristics comparison based on C-DII quartile groups (Q1–Q4).

Subgroup	Overall (*n* = 6,523)	Q1 (*n* = 1,631)	Q2 (*n* = 1,631)	Q3 (*n* = 1,630)	Q4 (*n* = 1,631)	*p*-value
Asthma						0.016
Yes	1,215 (18.63%)	308 (18.88%)	273 (16.74%)	308 (18.9%)	326 (19.99%)	
No	5,308 (81.37%)	1,323 (81.12%)	1,358 (83.26%)	1,322 (81.1%)	1,305 (80.01%)	
Gender						0.056
Female	3,299 (50.57%)	835 (51.20%)	799 (48.99%)	841 (51.60%)	824 (50.52%)	
Male	3,224 (49.43%)	796 (48.80%)	832 (51.01%)	789 (48.40%)	807 (49.48%)	
Age						0.281
6–9 years	1886 (28.91%)	460 (28.20%)	508 (31.15%)	445 (27.30%)	473 (29.00%)	
10–13 years	1944 (29.80%)	500 (30.66%)	465 (28.51%)	495 (30.37%)	484 (29.68%)	
14–16 years	1,441 (22.09%)	357 (21.89%)	328 (20.11%)	387 (23.74%)	369 (22.62%)	
17–19 years	1,252 (19.19%)	314 (19.25%)	330 (20.23%)	303 (18.59%)	305 (18.70%)	
Race/Ethnicity						0.001
Mexican American	1,322 (20.27%)	361 (22.13%)	364 (22.32%)	322 (19.75%)	275 (16.86%)	
Other Hispanic	689 (10.56%)	176 (10.79%)	172 (10.55%)	171 (10.49%)	170 (10.42%)	
Non-Hispanic White	1969 (30.19%)	487 (29.86%)	492 (30.17%)	523 (32.09%)	467 (28.63%)	
Non-Hispanic Black	1,468 (22.50%)	328 (20.11%)	340 (20.85%)	342 (20.98%)	458 (28.08%)	
Other races (including multi-racial)	1,075 (16.48%)	279 (17.11%)	263 (16.13%)	272 (16.69%)	261 (16.00%)	
PIR						0.009
< 1.3	2,414 (37.01%)	591 (36.24%)	570 (34.95%)	579 (35.52%)	674 (41.32%)	
1.3–3.5	2,816 (43.17%)	671 (41.14%)	726 (44.51%)	711 (43.62%)	708 (43.41%)	
> 3.5	1,293 (19.82%)	369 (22.62%)	335 (20.54%)	340 (20.86%)	249 (15.27%)	
BMI Categories						0.012
Underweight	73 (1.12%)	10 (0.61%)	14 (0.86%)	25 (1.53%)	24 (1.47%)	
Normal	3,080 (47.22%)	699 (42.86%)	777 (47.64%)	802 (49.20%)	802 (49.17%)	
Overweight	1,474 (22.59%)	403 (24.71%)	379 (23.24%)	359 (22.02%)	333 (20.42%)	
Obese	1896 (29.07%)	519 (31.82%)	461 (28.26%)	444 (27.24%)	472 (28.94%)	

Mean ± SD for continuous variables: the *p*-value was calculated by the weighted linear regression model. (%) for categorical variables: the *p*-value was calculated by the weighted chi-square test.

### 3.2 C-DII and asthma: overall analyses

#### 3.2.1 Logistic regression analysis

Building on a significant univariate association between asthma prevalence and C-DII quartiles observed in Table 1 (*p* = 0.016), the association between C-DII quartiles and asthma risk was evaluated through logistic regression, showing: **Quartile 2**: A significant protective effect (OR = 0.82, *p* = 0.034). **Quartile 3**: The protective effect weakened (OR = 0.94, *p* = 0.021). **Quartile 4**: Suggested an increased risk (OR = 1.01, *p* = 0.012). **Trend Analysis**: A significant graded relationship was observed across quartiles (*p* < 0.01; Table 2).

**Table 2:** Logistic regression analysis table.

Model	Model 1 OR (95% CI)	Model 1 *p*-value	Model 2 OR (95% CI)	Model 2 *p*-value	Model 3 OR (95% CI)	Model 3 *p*-value
Quartile 2	0.83 (0.82, 0.83)	0.034	0.82 (0.82, 0.82)	0.034	0.82 (0.82, 0.82)	0.034
Quartile 3	0.95 (0.95, 0.95)	0.021	0.95 (0.95, 0.95)	0.021	0.94 (0.94, 0.95)	0.021
Quartile 4	1.03 (1.03, 1.04)	0.012	1.03 (1.03, 1.03)	0.012	1.01 (1.01, 1.01)	0.012
P for trend	nan	0.001	nan	0.002	nan	0.003

The logistic regression models were adjusted for all covariates. Odds Ratios (OR) and 95% Confidence Intervals (CI) are reported. *p*-values were derived from the weighted regression models. Model 1: Unadjusted; Model 2: Adjusted for age and gender; Model 3: Adjusted for age, gender, ethnicity, BMI, and PIR.

#### 3.2.2 Inflection point and RCS analysis

**Figure 2:**
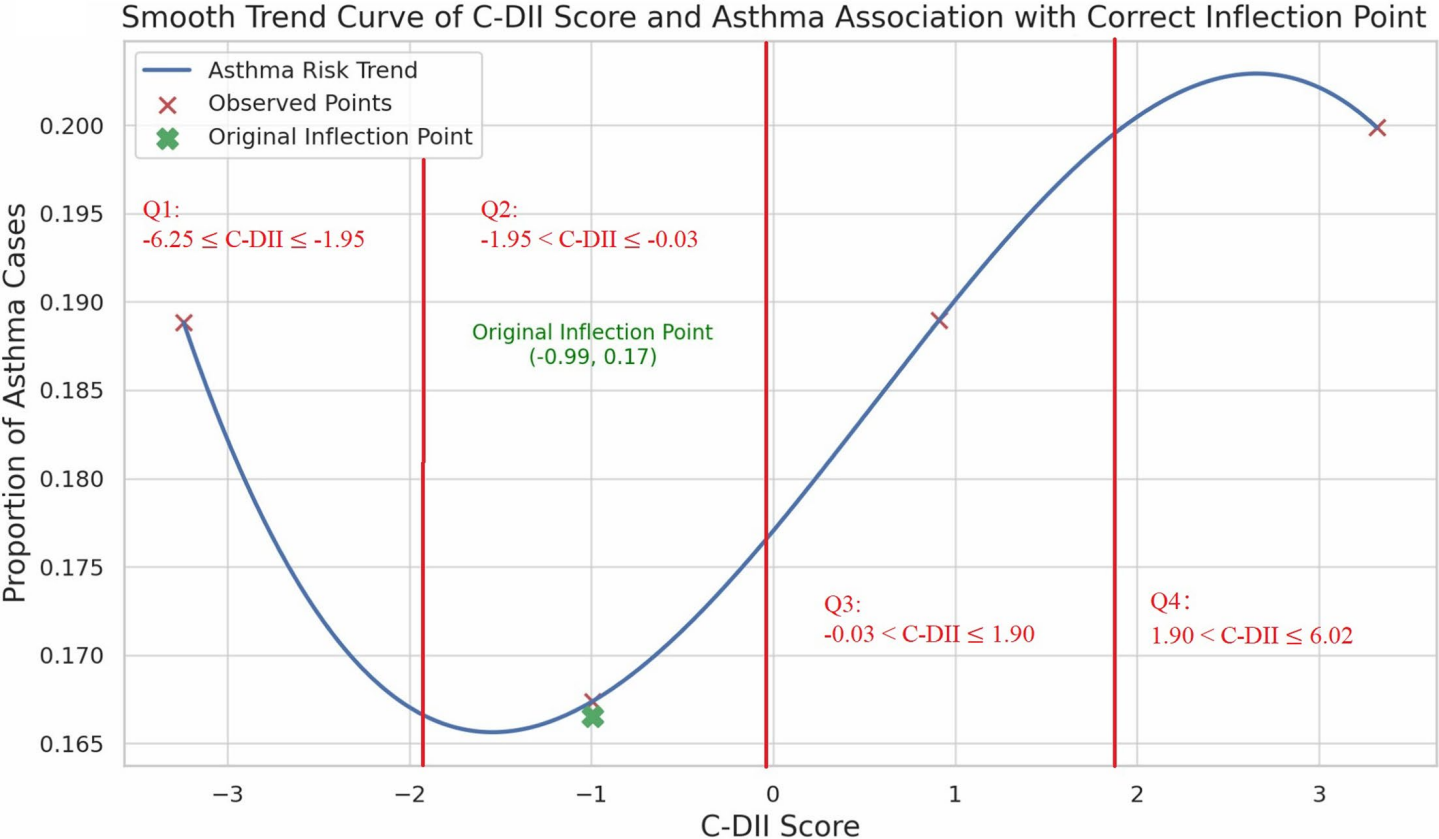
Smooth curve fitting showing U-shaped relationship between C-DII and asthma.

**Figure 3:**
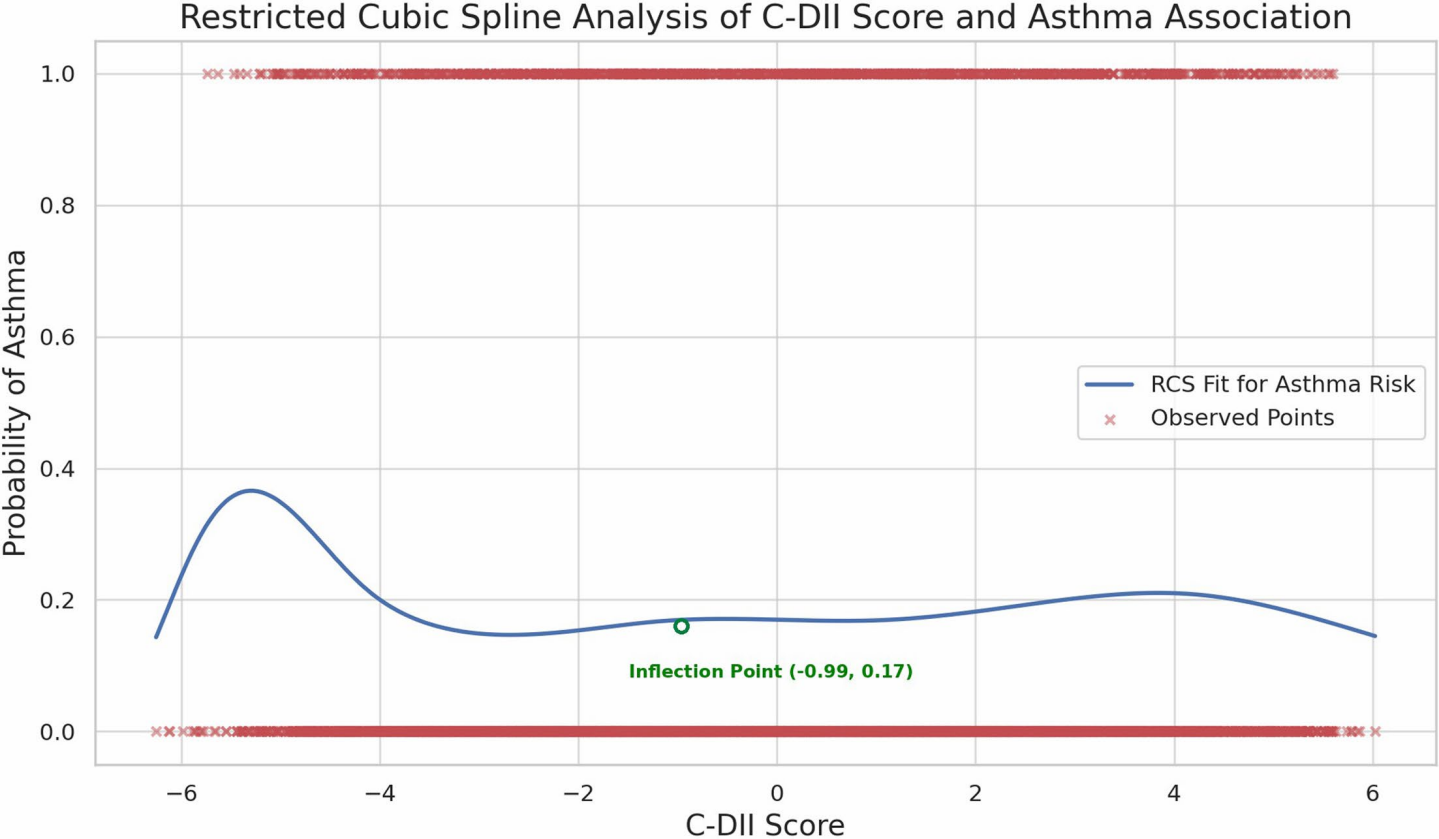
Restricted Cubic Spline (RCS) analysis of C-DII and asthma.

We performed smooth curve fitting and threshold benefit analysis on the results (Figure 2). The inflection point at **C-DII = −0.99** (in Quartile 2) indicated a change in the relationship pattern, with the lowest asthma risk around this value. Below this point, less inflammatory diets were associated with higher asthma risk. While above it, increased C-DII scores were also associated with greater asthma risk, revealing a U-shaped trend. The Restricted Cubic Spline (RCS) analysis confirmed this non-linear pattern, demonstrating a relatively stable risk at moderate C-DII scores, but an escalating risk at the extremes (Figure 3).

### 3.3 C-DII and asthma: subsidiary analyses and P for interaction

We assessed confounding by gender, age, ethnicity, PIR, and BMI, noting minimal impact on estimates (<2% change; Table 2), then tested interactions with C-DII to evaluate effect modification on asthma across these subgroups (Table 3).

**Table 3:** Subgroup analysis table.

Subgroup	OR (95% CI)	*p*-value	P for interaction
Gender			0.258
Female	0.88 (0.78, 1.00)	0.053	
Male	1.13 (1.00, 1.28)	0.053	
Age			0.047
6–9 years	1.05 (0.91, 1.20)	0.496	
10–13 years	1.07 (0.93, 1.22)	0.334	
14–16 years	1.00 (0.86, 1.16)	0.975	
17–19 years	0.86 (0.73,1.01)	0.061	
Race/Ethnicity			0.392
Mexican American	0.83 (0.70, 0.97)	0.021	
Other Hispanic	0.94 (0.77,1.16)	0.581	
Non-Hispanic White	0.89 (0.77,1.02)	0.086	
Non-Hispanic Black	1.56 (1.36,1.80)	0.000	
Other races (including multi-racial)	0.84 (0.70,1.00)	0.115	
PIR			0.961
< 1.3	1.23 (1.09,1.40)	0.001	
1.3–3.5	0.90 (0.79,1.02)	0.089	
> 3.5	0.88 (0.75,1.03)	0.029	
BMI Categories			0.049
Underweight	0.97 (0.68,1.29)	0.843	
Normal	0.99 (0.96,1.03)	0.847	
Overweight	0.96 (0.91,1.03)	0.108	
Obese	0.98 (0.94,1.06)	0.402	

Age, gender, race/ethnicity, poverty income ratio (PIR), and BMI were adjusted. In the subgroup analysis, the model is not adjusted for the stratification variable itself.

Gender: The interaction was not statistically significant (*p* = 0.258), with males (OR = 1.13, 95% CI: 1.00–1.28) and females (OR = 0.88, 95% CI: 0.78–1.00) showing slightly different point estimates for C-DII and asthma prevalence; however, the overlapping confidence intervals including 1.00 indicate no clear gender-specific effect.

Age: The age-based analysis was divided into four subgroups (6–9, 10–13, 14–16, 17–19 years), showing a significant interaction with C-DII (P for interaction = 0.047). For the 17–19 years group, the odds ratio was 0.86 (95% CI: 0.73–1.01, *p* = 0.061), which was not statistically significant. This suggests a potential trend toward a weaker association between C-DII and asthma in older adolescents, but the finding is not conclusive and warrants further exploration.

Ethnicity: Mexican-American (OR = 0.83, 95% CI: 0.70–0.97) and Non-Hispanic Black (OR = 1.56, 95% CI: 1.36–1.80) subgroups demonstrated significant associations compared to other ethnic groups (P for interaction = 0.392). These findings indicate that C-DII is associated with asthma risk in specific ethnic subgroups, but there is no clear evidence of differential effects across ethnicities. Variations in dietary patterns or genetic predispositions could be explored further, though the current data do not confirm an interaction by ethnicity.

PIR: No significant interaction was observed across poverty income ratio (PIR) groups (P for interaction = 0.961), suggesting that the effect of C-DII on asthma risk is not significantly modified by socioeconomic status overall. However, subgroup analysis revealed significant direct associations in specific PIR categories: PIR < 1.3 (OR = 1.23, 95% CI: 1.09–1.40) and PIR > 3.5 (OR = 0.88, 95% CI: 0.75–1.03). These findings indicate that C-DII may influence asthma risk in certain socioeconomic subgroups independently, despite the lack of a significant interaction across all PIR levels.

**Figure 4:**
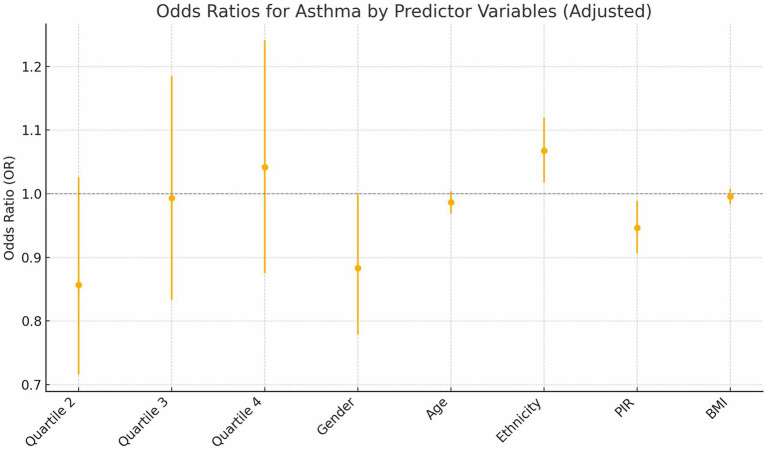
Adjusted odds ratios of asthma risk.

**Figure 5:**
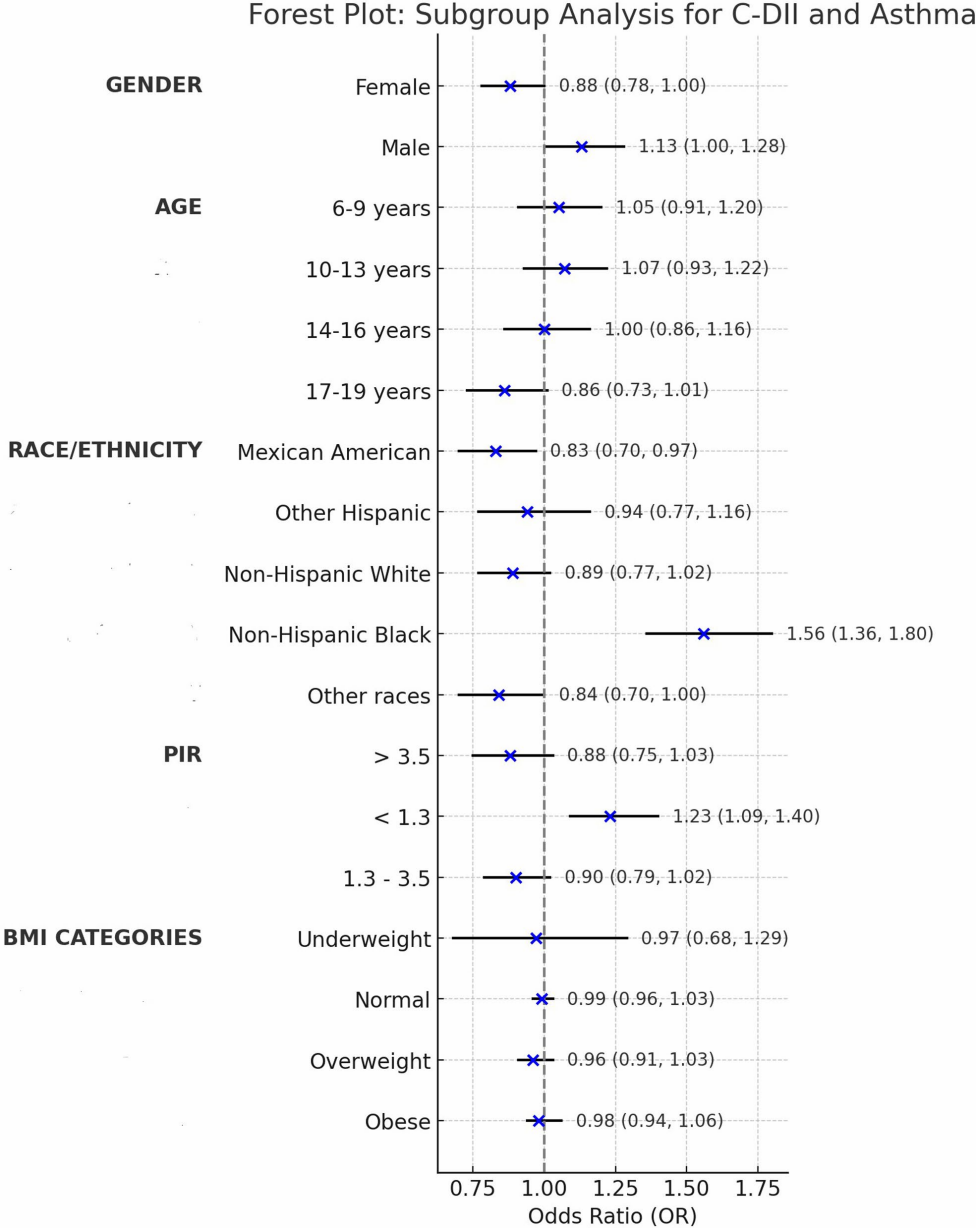
Forest plot of subgroup analysis.

BMI: Subgroup analyses revealed a significant interaction between BMI and C-DII in relation to asthma prevalence (P for interaction = 0.049). The odds ratios (OR) for asthma across BMI categories (underweight, normal weight, overweight, and obese) were not significantly different from 1, indicating no significant association between BMI and asthma prevalence. These results indicate that BMI did not significantly modify the association between C-DII and asthma (Figures 4, 5).

## 4 Discussion

In this study, we investigated the association between the C-DII and asthma prevalence among US children and adolescents, revealing a distinctive non-linear, U-shaped relationship. The inflection point at a C-DII score of −0.99 represents a critical threshold where dietary inflammatory potential shifts in its impact on asthma prevalence. Specifically, diets with moderate C-DII scores (near the inflection point) were associated with the lowest asthma risk, while both low and high C-DII scores correlated with increased asthma prevalence. While the OR in the 4th quartile (OR = 1.01) suggests a minimal increase in risk, it indicates that a pro-inflammatory diet may still contribute to asthma risk, albeit weakly.

The elevated asthma risk at extreme ends of the C-DII spectrum suggests that not only highly inflammatory diets, but also overly anti-inflammatory diets, may disrupt immune homeostasis and exacerbate susceptibility to asthma.

This U-shaped relationship contrasts with the predominantly linear associations observed in adult populations, where higher DII scores are generally linked to greater asthma risk (20, 21). The difference may be attributed to developmental and metabolic distinctions between adolescents and adults (22). Adolescents experience rapid physiological changes, including heightened metabolic demands and evolving immune systems, which could make them more sensitive to imbalances in dietary inflammatory potential (23). Furthermore, the interplay between dietary patterns and environmental exposures, such as allergens or air pollution, might be more pronounced during adolescence due to ongoing biological development (24). These results underscore the need for tailored dietary interventions that account for the unique physiological and environmental contexts of adolescents, emphasizing a balanced dietary approach to mitigate asthma risk. Our findings emphasize the importance of dietary balance during this critical developmental period. A moderate C-DII eating pattern, aligned with the inflection point of-0.99 identified in our results, reflects a balanced diet that includes both anti-inflammatory and nutrient-dense foods. Practically, this could mean a diet rich in fruits (e.g., berries, citrus), vegetables (e.g., leafy greens, broccoli), whole grains (e.g., quinoa, brown rice), lean proteins (e.g., fish, poultry), and healthy fats (e.g., nuts, olive oil, omega-3-rich fish like salmon), while limiting but not eliminating pro-inflammatory components like red meat or refined carbohydrates (25). This balance supports immune function and growth without tipping into extremes. Conversely, excessively anti-inflammatory diets—such as those overly reliant on low-calorie, plant-based foods (e.g., exclusive focus on leafy greens, fruits, or vegetable juices) while avoiding animal products or fats entirely—might lack essential nutrients like vitamin D (from fatty fish or fortified dairy), zinc (from meat or shellfish), and protein (from legumes or lean meats), which are critical for immune development and growth (26). For instance, a child on a highly restrictive vegan diet without supplementation could face these deficits. Similarly, diets high in pro-inflammatory components—such as frequent consumption of processed foods (e.g., fast food, sugary snacks), saturated fats (e.g., fried foods, butter), and refined sugars—can increase systemic inflammation, potentially worsening asthma symptoms (27). Recent studies have shown that ultra-processed foods, which contain additives and chemicals, are strongly linked to increased inflammation, which could contribute to asthma exacerbation (28).

These findings apply to both children and adolescents, highlighting the importance of balanced diets across these age groups.

Subgroup analyses revealed significant interactions between C-DII and key demographic variables, including gender, age, ethnicity, socioeconomic status, and BMI, underscoring the nuanced influence of dietary inflammation on asthma risk (Figure 5). Younger children demonstrated stronger associations between C-DII and asthma compared to older adolescents, possibly due to the immaturity of their immune systems, which may be more reactive to inflammatory dietary components (29). This heightened susceptibility during early developmental stages aligns with the critical role of nutrition in shaping immune and respiratory health during childhood.

The analysis showed no significant interaction between BMI and C-DII in relation to asthma. This may be due to the multifactorial nature of immune and inflammatory responses, which are influenced by genetics, dietary diversity, and environmental factors, potentially masking BMI’s effect (30). Additionally, BMI categories may oversimplify the complex relationship between body weight and inflammation, especially in children and adolescents, whose metabolic changes may not be fully captured by BMI classification (31).

Ethnic disparities were also evident, with Non-Hispanic Black populations experiencing stronger effects of dietary inflammation on asthma risk compared to other ethnic groups. These differences may reflect cultural dietary patterns, such as higher consumption of traditional foods with varying inflammatory potentials, or genetic predispositions influencing inflammatory responses (32). Socioeconomic status further modulated these associations, with individuals from lower-income households exhibiting a stronger link between C-DII and asthma (33). Limited access to anti-inflammatory foods, coupled with greater reliance on inexpensive, processed, pro-inflammatory diets, could contribute to this heightened risk.

Our findings align with previous research emphasizing the role of chronic low-grade inflammation in asthma pathogenesis, further supporting the biological plausibility of dietary components influencing systemic inflammation and, consequently, respiratory health. This study adds to the growing body of evidence by being one of the first to examine the relationship between C-DII and asthma in pediatric populations, providing critical insights into the dietary factors that may contribute to asthma prevention and management strategies.

There are several limitations to this study. First, as a cross-sectional study, it was not possible to establish a causal relationship between the Children’s Dietary Inflammatory Index (C-DII) and asthma prevalence. Secondly, the self-reported nature of asthma diagnosis in the NHANES dataset may introduce inaccuracies compared to clinical diagnoses, potentially leading to misclassification bias. And because of the exclusive reliance on 24-h dietary recall data, which may be subject to recall bias and may not fully represent habitual dietary intake. Thirdly, while we analyzed the relationship between C-DII and asthma, the NHANES dataset does not include information on asthma severity, such as the frequency or severity of exacerbations, limiting our ability to stratify asthma outcomes by severity. Furthermore, selection bias and recall bias cannot be entirely ruled out, as dietary intake and asthma status were self-reported. Another limitation is that this study focused on US adolescents, and the findings may not be generalizable to other populations with differing dietary patterns, environmental exposures, and genetic backgrounds. Finally, although we adjusted for key confounders such as age, sex, BMI, socioeconomic status, and ethnicity, residual confounding by unmeasured factors may still exist. For example, region—potentially influencing asthma prevalence due to variations in weather, air quality, or environmental factors—was not accounted for in this analysis. Due to the limitations of the NHANES dataset, data on medication use and physical activity were not included in the analysis.

Despite these limitations, this study has several strengths. To our knowledge, it is one of the first to investigate the relationship between C-DII and asthma in a pediatric population, providing novel insights into the role of dietary inflammation in asthma risk. The use of a nationally representative, multiethnic sample strengthens the generalizability of our findings within the US context. Moreover, the inclusion of a validated dietary inflammatory index tailored for children allows for a more nuanced understanding of dietary contributions to asthma risk (34, 35).

For future research, longitudinal studies are necessary to establish causal links between dietary inflammation and asthma. Exploring the relationship between C-DII and asthma across different severities and phenotypes, such as allergic and non-allergic asthma, would provide further clinical insights. Additionally, prospective studies in more diverse populations are essential to validate these findings and inform dietary guidelines for asthma prevention and management in children and adolescents.

## 5 Conclusion

This study highlights a significant, non-linear association between the C-DII and asthma risk in US children and adolescents, revealing a U-shaped relationship with the lowest risk at moderate C-DII scores. These findings underscore the importance of balanced dietary patterns in mitigating asthma risk. Subgroup analyses suggest that age and BMI significantly influence the C-DII–asthma relationship, while gender, ethnicity, and socioeconomic status show varying subgroup effects without significant interactions, suggesting the need for tailored dietary interventions targeting high-risk groups.

Despite its limitations, including the cross-sectional design and reliance on self-reported data, this study provides novel insights into the role of dietary inflammation in pediatric asthma. Future longitudinal studies are essential to confirm these findings, explore causal relationships, and develop evidence-based dietary guidelines for asthma prevention and management in children and adolescents. These results contribute to the growing understanding of how nutrition impacts respiratory health and emphasize the potential of dietary strategies as a component of asthma management.

## Data Availability

The raw data supporting the conclusions of this article will be made available by the authors, without undue reservation.

## Footnotes

^1^ https://www.cdc.gov/nchs/nhanes/
